# Incorporating Environmental Justice into Second Generation Indices of Multiple Deprivation: Lessons from the UK and Progress Internationally

**DOI:** 10.3390/ijerph13080750

**Published:** 2016-07-26

**Authors:** Jon Fairburn, Werner Maier, Matthias Braubach

**Affiliations:** 1 Business School, Staffordshire University, Staffordshire ST4 2DE, UK; 2 Institute of Health Economics and Health Care Management, Helmholtz Zentrum München, German Research Center for Environmental Health (GmbH), Neuherberg 85764, Germany; werner.maier@helmholtz-muenchen.de; 3 European Centre for Environment and Health, World Health Organization (WHO) Regional Office for Europe, Bonn 53113, Germany; mbr@ecehbonn.euro.who.int

**Keywords:** indices of multiple deprivation, environmental justice, geographic information systems, policy

## Abstract

Second generation area-based indices of multiple deprivation have been extensively used in the UK over the last 15 years. They resulted from significant developments in political, technical, and conceptual spheres for deprivation data. We review the parallel development of environmental justice research and how and when environmental data was incorporated into these indices. We explain the transfer of these methods from the UK to Germany and assess the progress internationally in developing such indices. Finally, we illustrate how billions of pounds in the UK was allocated by using these tools to tackle neighbourhood deprivation and environmental justice to address the determinants of health.

## 1. Introduction

Since the year 2000, there has been a significant increase in the use of second generation area-based multiple deprivation indices in public policy and research in the UK. Academics first used these indices to examine environmental justice and then also led on incorporating environmental data into later versions of these indices. This work not only spurred further research and development in environmental justice [1] but also directly fed into public policy [2], especially public policy that would have a direct impact on the determinants of health.

Deprivation is the absence of something considered to be a necessity for a person to function or take part in society; this could be a material factor such as a house or income, or a job or good health. Deprivation can be an absolute or relative phenomenon and both perspectives tend to be used in policy and research [3].

Environmental justice has two aspects to it: distributive justice, i.e., which populations experience good environments and which experience poor environments (examples include [4,5]); and procedural justice, which involves access to information and the decision making process (e.g., [6]). In some instances, distributional studies have informed or led to changes in procedural justice. In other cases, distributional studies have been occasioned by procedural concerns.

At the outset, environmental justice in the UK concerned itself with a very different set of factors than had been the focus in the USA [7]. Whilst researchers in the USA focused mainly on race, scientists in the UK started with income as the dependent variable and quickly moved to using general measures of deprivation and the creation of neighbourhood data. Furthermore, the environmental factors considered in the UK (indicators for both good and bad environments) were also much broader in scope than those that had been used in the USA.

We begin the paper by examining the technical, conceptual, and political factors that were successfully overcome to develop Indices of Multiple Deprivation (IMD) in the UK and how environmental justice results were incorporated into them. We then show how the lessons from IMD creation in the UK were transferred to Germany to provide their first index of multiple deprivation (including environmental information). We provide an international assessment of a number of countries’ current status in developing such indices and where environmental data has been included. Finally, we provide evidence of how billions of pounds were allocated in the UK according to such indices, which directly or indirectly attempted to tackle environmental justice, neighbourhood quality, and address the determinants of health.

## 2. Transformations in the Political, Conceptual, and Technical Use of Official Data

The UK has been at the forefront in the development and use of Indices of Multiple Deprivation. Several first generation indices were created in the 1970s, 1980s, and 1990s including the Townsend Index [8], the Carstairs Index [9], the Jarman Score [10], and the Breadline Britain Index [11]. These indices were census based and characterised by a small number of variables, some of which were proxies for deprivation (e.g., car ownership levels).

The second generation of indices in the UK was driven by a stated New Labour government demand for evidence based policy linked to a progressive political agenda to tackle deprivation. The creation of these new Indices of Multiple Deprivation (IMD) from 2000 onwards [3] and the progress made in environmental justice distributional studies was achieved by a mix of political, conceptual, data, and technical factors including the following:

1 Change in official attitudes to the sharing and availability of state held official data. Such data was also more up to date than the census data previously used. This was accompanied by the development of domains that grouped individual indicators together to classify different experiences of deprivation.
2 Creation and then availability and access of digital spatial boundary data sets at the level of the small area unit. These were often at a finer spatial resolution than had traditionally been available.
3 Tagging of socio-economic data with spatial information so that different datasets could be merged and new information created, usually in a Geographic Information System (GIS) environment.
4 A key development was the move away from census proxy indicators to government benefits data and other direct measures of deprivation.
5 Complete coverage of the population (100%) as opposed to sample survey methods.
6 Time series of data to monitor change over time.
7 Commission and adoption of the Indices of Multiple Deprivation as an official policy tool by government and their agencies.

Each country in the UK has their own version of the IMD (see Table 1), with small variations occurring due to availability of particular datasets, the outcomes of consultation or the time when the index was created. Indices are dominated, i.e., heavier weighted by employment and income domains, but other domains can include health, crime, environment, and proximity to services. The importance of thinking of deprivation in multiple domains is because an individual domain of deprivation may be ameliorated or amplified by another domain.

The creation of these indices has been described [12,13,14,15,16] and an early critique of the English IMD made [17]. More recently, Payne and Abel 2012 have suggested it would be possible to create an IMD for the whole of the UK [18] using the existing data.

The DETR (2000) report [12] found that there were no available indicators for the physical environment at that time that could be part of the first English and Welsh IMD. The main reasons were the lack of suitable data, although in one case it was the result of an inability to access the information. However, the results of Walker and colleagues [19] for the Environment Agency have led to the inclusion of air quality measures in all subsequent indices in England. This was an especially important development, as they showed very strong levels of inequality for air quality levels with poor areas experiencing high numbers of people exposed to air quality above the safety limits. Since that study, it has consistently been shown that poor communities experience the worst air quality, which has a direct impact on mortality and morbidity rates, and this led to the development of policy to address the issue [20]. The environmental aspects of the later indices in England consisted of an outdoor domain and an indoor domain. In the outdoor domain, the incidence of road traffic accidents was the other variable alongside the air quality metric. In the indoor domain, poor housing quality (a recognised standard) and lack of central heating were the two components used.

In Wales, the 2005 IMD [21] went even further than the English IMD by incorporating all the environmental factors that had been identified in the Walker et al. study [19], namely: air quality; proportion of people living within 1km of emission sites; and proportion of people at risk of flooding.

In Scotland there was an intention to include physical environment data in the original IMD with the initial report stating “introduction of the physical environment domain (see report, section R5.1.8). There are links to developing measures of environmental justice which should be considered” [22]. The Scottish Government, Scottish Environmental Protection Agency, and Scottish Natural Heritage and Forestry Scotland all co-operated to fund the SNIFFER report [4]. Despite this report showing that environmental inequality was present amongst the population for several environmental quality measures, environmental factors have never been incorporated into the Scottish Index of Multiple Deprivation. However, the production of the SNIFFER report did influence the creation of the Forward Scotland fund (£2 million) to support deprived communities that had suffered negative environmental impacts.

## 3. Transferring the Lessons from the UK to Germany

Area deprivation indices are integral parts of the public health discussion in the United Kingdom. In Germany, however, the discussion on this topic only began in the 2010s. Based on an established British method as described by Noble et al. [3] and equipped with the experience of a research stay in the UK, Maier and colleagues developed an Index of Multiple Deprivation for Germany. Starting with the model region of the German federal state of Bavaria and using official sociodemographic, socioeconomic, and environmental data, they created a *Bavarian Index of Multiple Deprivation* (BIMD) [30]. Using the BIMD on the level of municipalities, Maier and colleagues could show that higher regional deprivation is associated with higher mortality in Bavaria and stated that this index is a useful tool for epidemiological and public health related studies also in Germany. They could demonstrate the transferability of the British IMDs outside the UK by adapting it to the German context. In a next step, Maier and colleagues created a nation-wide IMD, the *German Index of Multiple Deprivation* (GIMD). They could show that the prevalence of the type 2 diabetes is associated with area deprivation on the municipality as well as on the district level [31,32]. Meanwhile, the German indices have been used in a number of publications in the fields of epidemiology, e.g., cancer or diabetes [33,34,35], of health services research, e.g., hip and knee joint replacement or antibiotic prescriptions [36,37], health economics [38], and environmental justice [39].

In a relatively short period, the GIMD received some attention from health policy and has been mentioned in expert reports of the Advisory Council on the Assessment of Developments in the Health Care System appointed by the German Ministry of Health [40].

However, the federal structure of Germany provides a challenging task to constructing such an index and there are several limitations of the GIMD to be mentioned. There are only a limited number of available variables at municipality level which is the smallest area level available in official statistics in Germany. Moreover, municipalities cover a wide range of population sizes, including small rural communities with less than 100 inhabitants up to cities with more than one million inhabitants, e.g., Berlin [31]. However, considering their methodological approach based on the work of Noble and colleagues [3], the German deprivation indices are certainly the closest ones to the UK indices of multiple deprivation. The current update of the GIMD as well as the plans for its extension regarding indicators and domains provide hope that this index will not be a short-term phenomenon.

## 4. Current International Progress on Developing Indices of Multiple Deprivation

Pasetto et al. [41] provide a review of deprivation indices between 1990 and 2009. All 41 of the studies cited can be considered first generation indices and clearly indicate the limitation of this approach. Apart from the common use of census data that are proxies (e.g., lack of car ownership, rental levels), the biggest problem is that—at best—these are historical studies. Commonly the census data used is over a decade old, furthermore although in many of the cases they have managed to overlap the health and environmental data the datasets do not always align in the same years. Probably the biggest problem is that the data used in these studies is so out of date that it does not easily lend itself to current spatial and planning policy decisions.

The need to measure equity has been recognised at an international level [42,43], however, relatively few countries currently manage to do this effectively and consistently. Furthermore, consistent international comparison across countries using deprivation indices is a very long way from satisfactory. A working group of international experts convened to prepare a WHO assessment of environmental health inequalities in the European Region [44] found it was only possible to compare countries on the basis of survey data on self-reported environmental conditions, rather than objective measures of environmental exposure. There is little objectively observed spatially tagged data that is consistently recorded across countries that can be used to create an index of deprivation for a group of countries, although some have tried.

Table 2 provides a selection of Indices of Deprivation within Europe. The general picture is that there is in general no or little official use of such indices, little and usually no incorporation of environmental information into the index, some development of domains and indicators, and some use of them in environmental justice research. These studies are also often a single occurrence with no later updating of the index. However, some countries, e.g., France, Germany, and Italy show repeated attempts in the creation and the use of deprivation indices.

## 5. General Barriers to Creating an Index of Multiple Deprivation

There are many barriers to be overcome before a country index of deprivation can be created. These include the following data or technical reasons:

1 Attribute data does not exist for the whole population other than the census (e.g., many countries use a sample survey approach).
2 Data are only census-based and become out of date (Germany, for example, rarely conducts a census; although, updated statistical information is available in Germany on a yearly basis).
3 Attribute data do exist but are not publically available, this is usually the default position for government datasets such as benefits. Measures have to be put in place to maintain confidentiality.
4 Attribute data are publically available but not in digital format or are in a digital format (e.g., PDF) but do not readily lend themselves to use in databases or GIS.
5 Data are not collected at a consistent small unit area level other than maybe the census. Or data is tied to different spatial units and cannot be easily merged to create new data.
6 Digital datasets for spatial unit boundaries do not exist.
7 Digital datasets for spatial unit boundaries exist but are restricted or prohibitively expensive.
8 No easily available identifier to link the spatial data to the attribute data.

However, with the increasing transformation of data collection through digital means plus the general drive to a digital society, technical issues are no longer the barrier to creating such indices. When emerging economies such as South Africa [63] and Nambia [64] are using indices of multiple deprivation, the question needs to be asked as to why relatively few countries in Europe and elsewhere do?

Of course underlying the development and use of indices as a public policy tool are the political and cultural values of the country under consideration and the existing tools and methods used in public policy. There may be a reluctance to change or develop a new approach due to the invested expense, longevity, and familiarity of existing sources and tools. Furthermore, national frameworks and regulations may be implemented differently in different parts of a given country, especially when regions or federal states have an independent mandate for implementation and as such a consistent national dataset may not be possible. However, with the rise of poverty as a key point of debate in social policy on national and international level, governments may also be less eager to document rising equity gaps in the population. Such lack of interest could be enhanced by the perception of social equity as a target impossible to achieve even in times of economic growth [65].

Civil servants can be enormously reluctant to release data. Traditionally, the UK was very hesitant in releasing data but now there are Open Government initiatives [66] to increase transparency and to encourage the re-use of data in the UK [67]. In Germany, a profound public scepticism to sharing private data, which may be explained by historical experience has meant a reluctance to carry out population censuses. Before the census in 2011, the last one was held in 1987. Germany has relied on regularly updated administrative data and on large surveys such as EU-SILC (European Union Statistics on Income and Living Conditions) from, the KiGGS or the GEDA surveys, both conducted by the Robert Koch Institute [68].

Environmental data is supposed to be available under the Aarhaus Convention (1998) UNECE in many countries, although it has sometimes required legal threats by NGOs and others to get access. Work to increase access to environmental information continues under the UNECE (2015) [69].

To help evaluate the usefulness of area-based indices we can consider the advantages and disadvantages of such an approach.

### 5.1. The Advantages of Area-Based Indices

1 The Index of Multiple Deprivation can be presented and analysed using a GIS mapping tool. Maps are a very powerful communication device and are commonly understood and used by politicians and the general public. For example, in the UK the use of online mapping sites [70] and data tools [71,72] readily communicates information to the general public as everyone knows their postcode. The postcode in the UK is used as a unique identifier to bring together different datasets and forms the building block for socio-economic data.
2 The mapping of indices allows the targeting of resources to address a problem. In Scotland, for example, access to woodland for deprived groups was increased by targeting grants for new woods at the most deprived areas using a spatial approach with a GIS by Forestry Commission Scotland.
3 It is possible to map change over time. This is a particularly important point as the IMD can provide a baseline of data both before and after the interventions and so be used to assess the effectiveness of public policy interventions.
4 Planning policy at the regional or local level is one of the strongest means for effecting change. This can be through the granting or refusal of licences for activities with potential negative impacts, through the redesign of urban space which could improve environmental quality (pedestrianisation or restriction of traffic) through compensation for new developments in the provision of green space. A spatial approach ties directly to the planning system, which allows discussion of both the distributive aspects of environmental justice and the procedural ones. This approach is currently out to consultation with specific reference to environmental justice and the planning system by the Scottish Government which includes the proposal to create an all Scotland environmental court [73].

### 5.2. Disadvantages of Area-Based Indices

1. The ecological fallacy—these are deprived areas, not deprived individuals. Not all individuals who live in a deprived area are deprived and not all deprived individuals live in deprived areas. This means that they do not identify any vulnerability differential between populations living within the selected area. This is a longstanding and well-known issue in the discipline [74,75].
2. Methodological issues regarding the weighting of different comments of the index has been raised as an issue. In the most recent IMD 2015 the construction of the index was subject to a two stage consultation process which included discussion of this aspect. No technique is ever neutral especially when dealing with deprivation. Debate and discussion of the domain weightings and transparency of methods are needed and can increase confidence in these tools.
3. Large indices draw from various variables representing various dimensions, and are very useful for an overview purpose, but at the same time they may be hiding detailed data and indications—the more variables from different dimensions are included, the more abstract the result is.

## 6. Use of the Indices of Multiple Deprivation in UK Public Policy

Both directly and indirectly, environmental justice research has impacted on public policy through the incorporation of environmental data into Indices of Deprivation in England, Wales, and Northern Ireland. Such Indices can be considered superior to first generation indices as they represent the intersection of economic, social, and environmental factors which, together, provide a holistic measure of the neighbourhood, which can be linked to health outcomes based on timely and accurate data.

The IMD in the UK has been and is extensively used by local authorities, health organisations, charities, funders, and central government to spatially target areas for intervention and funding. The UK government for example has used it to automatically delineate which local authorities would be eligible for funding under different programmes. For example, the New Deal for Communities was a 10-year programme restricted to local authorities ranked in the bottom 15% of the country by the IMD (and was worth approximately £50 million per partnership to transform these neighbourhoods). The programme used an approach of tackling deprivation on many fronts, e.g., job search and training; housing; environment; crime; and neighbourhood improvement [76].

The Neighbourhood Renewal fund (which was just one instrument of government) was spending over £500 million per year (between 2001 and 2011) using the IMD to target areas for help with children’s centres, nurseries, and other neighbourhood interventions. A large part of the budget (£430 million in 2002–2003) for Regional Development Authorities was also allocated using the IMD to support economic development (see Oxford University for more examples [77]). A St Andrews University submission [78] estimated that 1% of government spending ca. *£7 billion per year* was being allocated using the IMD, most of which can be considered to have been targeted directly or indirectly at the determinants of health and deprivation.

It is not the aim of this paper to provide a systematic review of case studies that have used the IMD, but it is worth noting some of these to demonstrate the wide range of their use in research, some key findings and impact on policy.

Cummins et al. [79] found a statistically significant increase in the number of McDonald restaurants in Scotland in deprived areas positing an environmental explanation for higher obesity levels in these areas. This and other studies lead to many local authorities to introduce a ban on new takeaway food outlets in many parts of the country. For example, the London Borough of Waltham Forest policy states “the council will not give planning permission to new hot food takeaways if they are 400 m or less from a school, youth facility, or park. The policy aims to limit the opportunities that young people have to eat “fast food”, thus reducing childhood obesity”.

Major health events in the UK have been shown to have a socio-economic spatial component. For life expectancy Woods et al. [80] found that deprivation could explain life expectancy patterns in England and Wales using the first IMD. Woodward et al. [81] used the Scottish IMD to develop a more accurate cardiovascular risk indicator as classic cardiovascular risks scores do not reflect the social variation in the disease. Howieson ([82], p. 18) used the SIMD to look at winter deaths in Scotland and found “the majority of excess winter deaths are premature and essentially preventable if the elderly can be kept warm in their homes during the winter months”.

Access to green space is another example of how the UK has differed from the USA when considering which factors to assess in environmental justice. American studies have tended to focus on environmental factors that may cause or are perceived to cause harm to health. In the UK there was also a great deal of work on access to good environments [83], especially for recreation to quality green space and woodland areas. For example, Making the Links: Greenspace and the Partnership Agreement (2004) produced by Greenspace Scotland in partnership with Communities Scotland, NHS Health Scotland and Scottish Natural Heritage [84] with the aim of highlighting the value of green space to the economic, social, and environmental well-being of communities.

Government policy on forestry organisations (e.g., Scottish Forestry Strategy 2006 [85]) and green space organisations implemented a general policy to open up access to areas as well as programmes that targeted specific social groups using an environmental justice approach.

## 7. Conclusions

The UK has experienced a transformation in the use of small area data to develop and implement a second generation of Indices of Multiple Deprivation. These have had a very significant impact on government policy in multiple ways to address populations experiencing multiple deprivation (including environmental quality) and thus some of the key determinants of health.

Technical and data barriers can demonstrably be overcome if there is the political will to change and a recognition of the enormous benefits to providing comprehensive data to researchers, policy makers, politicians, and other users. The value of online mapping tools can help prepare the public for environmental threats (e.g., warnings of flood areas or poor air quality) as well as to inform decisions and debate on local issues.

Even where environmental justice was not incorporated into the Scottish Index of Multiple Deprivation, distribution studies did occur and were influential in changing policy and informing debate. The relevance of environmental justice continues with cross party political support in Scotland as shown by the current consultation on environmental justice and in the Labour Party a spokeswoman on Environmental Justice Sarah Boyack MSP.

Progress in developing second generation indices of deprivation outside the UK remains frustratingly slow as shown by Table 2, although working together internationally we have managed to transfer some of the lessons from the UK to Germany. We urge other researchers to push for the opening up of government held data and for the development of more second generation indices as a tool of public policy.

## Figures and Tables

**Table 1:** Indices of Multiple Deprivation in the UK (given information is based on the most recent version).

Index	Country	Year of First Version	Year of Recent Version	Number of Domains/Indicators	Smallest Spatial Unit	Average Population per Spatial Unit
English Indices of Deprivation (ID, IMD)	England	2000 [23]	2015 [16]	7/37	Lower-layer Super Output Area (LSOA)	ca. 1500 people
Scottish Index of Multiple Deprivation (SIMD)	Scotland	2004 [24]	2012 [25]	7/38	Datazones	ca. 800 people
Welsh Index of Multiple Deprivation (WIMD)	Wales	2000 [26]	2014 [27]	8/33	Lower layer Super Output Area (LSOA)	1600 people (min. 1000)
Northern Ireland Multiple Deprivation Measure (NIMDM)	Northern Ireland	2001 [28]	2010 [29]	7/52	Super Output Area	2000 people

The IMD in the UK is usually released at two levels of spatial unit: a smaller unit (super output area or datazone); and then the local authority level.

**Table 2:** Selection of Area Deprivation Indices in Europe.

Country	Index Denomination Reference	Population per Unit	Indicators: N (Total)/ N (Environment)	Data Source Updates Official Use Environmental Justice Application (EJ) Other Country-Specific Deprivation Indices (DI) Comments
Belgium	- Deprivation index - Lorant, 2000 [45]	- Community - Pop. not specified	11/-	- Data source: Census data and administrative (tax) data - No information on updates, official use, EJ
Czech Republic	- Socio-economic deprivation index (SESDI) - Šlachtová et al., 2009 [46]	- Enumeration districts - Max. 400 people	9/-	- Data source: Census data - No information on updates, official use, EJ
Denmark	- Danish Deprivation Index (DANDEX) - Meijer et al., 2013 [47]	- Parish - Median: 2545 people	9/-	- Data source: administrative data (presum.) - No information on updates, official use, EJ
France	- Small-area index of socio-economic deprivation - Havard et al., 2008 [48]	- Census block (IRIS) - Average of 2000 people	19/-	- Data source: Census data - Update: Lalloué et al., 2013 [49] - No information on official use - EJ: Air pollution (Laurent et al., 2008 [50]; Padilla et al., 2013 [51], 2014 [52]) - Other DI: - FDep99 (Rey et al., 2009 [53]) - EDI (Pornet et al., 2012 [54]; Guillaume et al., 2016 [55])
Germany	- German Index of Multiple Deprivation (GIMD) - Maier et al., 2013 [31]	- Municipality - Median: 2241 people	9/1	- Data source: administrative data - Recent update, manuscript in preparation - No official use, but increasing awareness of local/health authorities - EJ: Neighbourhood Greenness [39] - Other DI: Bavarian Index of Multiple Deprivation, BIMD, Maier et al., 2012 [30] - Comments: BIMD served as a model for the German DI (GIMD); Methodological approach cf. UK
Hungary	- Hungarian deprivation index - Juhász et al., 2010 [56]	- Municipality (LAU2) - Mean: 3253 people	7/-	- Census data and administrative (tax) data - No information on updates, official use, EJ
Italy	- Italian deprivation index - Caranci et al., 2010 [57]	- Census block - Average of 169 people	5/-	- Data Source: Census data - No information on updates, official use, EJ - Other DI: - 2 DI of material and social deprivation (Testi & Ivaldi, 2009 [58]) - ad hoc DI of SENTIERI project [59] for contaminated sites [60]
Spain	- Deprivation index for small areas - Sánchez-Cantalejo et al., 2008 [61]	- Municipality - Average of 5204 people	6/-	- Data source: Census data - No information on updates, official use, EJ
Switzerland	- Swiss neighborhood index of socio-economic position (Swiss-SEP) - Panczak et al., 2012 [62]	- Neighbourhood - ca. 50 households	4/-	- Data source: Census data - No information on updates, official use, EJ
